# A Rare and Lethal Complication of Oral Warfarin Therapy

**DOI:** 10.5005/jp-journals-10071-23156

**Published:** 2019-04

**Authors:** Subodh Kumar Mahto, Ankita Sheoran, Nagina Agarwal, Pooja Verma, Atul Goel

**Affiliations:** 1–3 Department of Medicine, PGIMER, Dr RML Hospital, New Delhi, India; 4,5 Department Medicine Smt. Sucheta Kriplani Hospital and Lady Hardinge Medical College, New Delhi, India

## Abstract

**How to cite this article:** Mahto SK, Sheoran A, *et al*. A Rare and Lethal Complication of Oral Warfarin Therapy. Indian J Crit Care Med 2019;23(4):197–198.

Sir,

A 58-year-old man presented to medicine emergency with a painless red swelling below the tongue causing difficulty in speech and swallowing for one day. He had been on oral warfarin (5 mg/day) for atrial fibrillation for past 6 months. His international normalized ratio (INR) was monitored weekly with a target of 2–3. There was no history of recent trauma or bleeding from any site. On examination, the patient was conscious and oriented. His pulse rate and blood pressure were 104/min and 120/80 mm Hg, respectively. Local examination revealed a tense, tender, red submucosal hematoma involving the floor of the mouth, and ventral lingual surface across the midline (Figs 1 to 3). The tongue was pushed slightly upward and the patient could protrude his tongue only with difficulty. Rest of general and systemic examination revealed no obvious abnormality.

Blood counts, liver, and kidney function test were within normal limits. However, the INR at presentation was high (5.6). Flexible endoscopic examination revealed that there was no extension of the swelling into the pharynx, laryngeal mobility was normal and the airway was not compromised. The patient was diagnosed with clinical findings that he had sublingual hemorrhage caused by excessive dose of warfarin. Since there were no signs of impending airway compromise; he was managed conservatively with vitamin K (5 mg intravenously for 3 days) and 4 units of fresh frozen plasma. Warfarin was discontinued, and the INR returned to normal within 48 h. The patient recovered clinically, and was discharged on the 4th day. The patient was then put on dabigatran (110 mg orally, twice daily).

**Figs 1 and 2:**
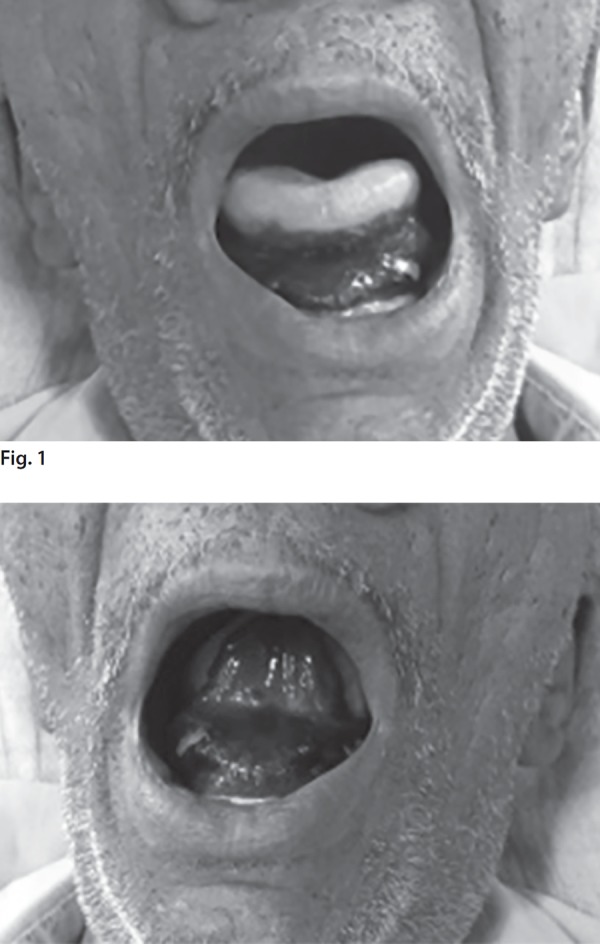
Red submucosal hematoma involving the floor of the mouth and ventral lingual surface across the midline.

**Fig. 3:**
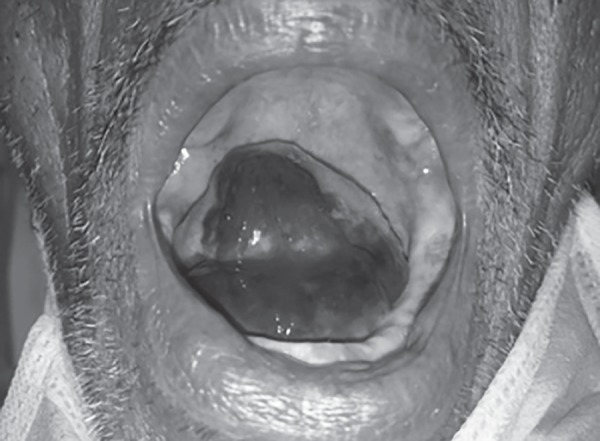
Submucosal hematoma involving the ventral surface and right lateral border of the tongue.

Warfarin is widely used in prophylaxis and treatment of thromboembolic events.^1^ Bleeding is the most common side effect of warfarin therapy. Bleeding complications of warfarin have typically been seen in intracerebral, skin, gastrointestinal system, genitourinary system, adrenal glands, epidural space, peritoneal cavity, and subconjuntival area. However, sublingual hemorrhage is extremely rare but potentially fatal complication of oral warfarin therapy.^1–3^ It is important to differentiate this condition from infectious processes such as Ludwig's angina as they are managed differently.^2^ Medical therapy with reversal of the coagulopathy with vitamin K, fresh frozen plasma, or factor concentrates remains the mainstay of management in the absence of airway compromise, which if present may necessitate an artificial airway.^2^ With an expanding hematoma, elevation of the tongue and floor of mouth can cause airway obstruction. In these cases, laryngoscopic intubation is difficult. Early definitive airway stabilization should be the priority with rapid sequence intubation. If rapid sequence intubation fails, emergency cricothyroidotomy or tracheostomy may be performed for securing definitive airway in the emergency department^4^Patient and his/her relatives should be educated about the side effects of the oral anticoagulant drugs. Sublingual hematoma is a very rare and lethal complication of anticoagulant drugs due to potential airway compromise. However, early diagnosis and prompt treatment only can prevent lethal complications, as life-threatening conditions including airway obstruction and asphyxia develop very fast in such cases.
